# Supplementary material for: Integrable Magnetic Fluid Hyperthermia Systems for 3D Magnetic Particle Imaging
Source: Nanotheranostics. 2024 Feb 12;8(2):163–78. doi: 10.7150/ntno.90360 (PMC10911971; doi:10.7150/ntno.90360)
Supplement: Supplementary file 1 — Supplementary derivations, figures and tables. [file ntnov08p0163s1.pdf]

Supplementary Materials

S1 - Derivation of matching factor

For an unmatched coil the RMS current through an electromagnetic coil is:

$$|I_{\mathrm{test}}| = \frac{S_{TX}}{|Z^*|} = \sqrt{\frac{P_{\mathrm{TX}}}{|Z|}} = \sqrt{\frac{P_{\mathrm{TX}}}{\sqrt{R_{\mathrm{L}}^2 + (\omega L)^2}}}$$

where S_{TX} is the complex power transmitted by the coil which in this case is equal to the amplifier power P_{TX}, R_L is the resistance of the coil,ω the angular frequency, L the inductance of the coil and t the time. For a matched coil, for instance using an impedance matching, the RMS current is calculated by:

$$|I_{\mathrm{heat}}| = \sqrt{\frac{P_{\mathrm{TX}}}{R_{\mathrm{L}}}}$$

The induced voltage in a coil due to an electromagnetic field is given by Faraday's law of induction and depends on the time-derivative of the magnetic field. The magnetic field depends on the geometry of the coil ensemble as well as the field generating current. Since the geometry is assumed not to change it can be modeled as a factor σ. The induced voltage then depends solely on the time-derivative of the field generating current. The field-generating current is a sinusoidal current with fixed angular frequency ω, hence:

$$U_{\mathrm{testRX}} = -\sigma \frac{\partial}{\partial t}(|I_{\mathrm{test}}| \cdot \sin(\omega t))$$

$$U_{\mathrm{heatRX}} = -\sigma \frac{\partial}{\partial t}(|I_{\mathrm{heat}}| \cdot \sin(\omega t))$$

$$\Rightarrow \frac{U_{\mathrm{testRX}}}{U_{\mathrm{heatRX}}} = \frac{|I_{\mathrm{test}}|}{|I_{\mathrm{heat}}|} \frac{\frac{\partial}{\partial t}(\sin(\omega t))}{\frac{\partial}{\partial t}(\sin(\omega t))} = \frac{|I_{\mathrm{test}}|}{|I_{\mathrm{heat}}|}$$

where U_{testRX} and U_{heatRX} are the voltages induced in the scanner from the test coil and the heating insert, respectively. The ratio of the powers transmitted to the coil can then be calculated as:

$$\frac{P_{\mathrm{testRX}}}{P_{\mathrm{heatRX}}} = \frac{U_{\mathrm{testRX}}^2}{Z_{\mathrm{MPI}}} \frac{Z_{\mathrm{MPI}}}{U_{\mathrm{heatRX}}^2} = \frac{U_{\mathrm{testRX}}^2}{U_{\mathrm{heatRX}}^2} = \frac{|I_{test}|^2}{|\mathrm{I}_{\mathrm{heat}}|^2} = \sqrt{\frac{P}{\sqrt{R_{\mathrm{L}}^2 + (\omega L)^2}}}^2 \sqrt{\frac{R_{\mathrm{L}}}{P}}^2 = \frac{R_{\mathrm{L}}}{\sqrt{R_{\mathrm{L}}^2 + (\omega L)^2}}$$

Finally, the correction factor for the transfer-functions is given by

$$\frac{P_{\mathrm{heatRX}}}{P_{\mathrm{TX}}} = \frac{P_{\mathrm{testRX}}}{P_{\mathrm{TX}}} \frac{\sqrt{R_{\mathrm{L}}^2 + (\omega L)^2}}{R_{\mathrm{L}}}$$

S2 - Derivation of power level and voltage level relation

The complex power of a coil can be expressed as:

$$S = \frac{|U|^2}{Z^*}.$$

Thus the apparent power is:

$$|S| = \left|\frac{|U|^2}{Z^*}\right| = \frac{|U|^2}{|Z^*|} = \frac{|U|^2}{|Z|}.$$

For the MPI system and the MFH system, this yields:

$$|S_{MPI}| = \frac{|U_{MPI}|^2}{|Z_{MPI}|}$$

and

$$|S_{MFH}| = \frac{|U_{MFH}|^2}{|Z_{MFH}|}$$

The transfer function measurements give the logarithmic magnitude of the power level, which is expressed as:

$$L_P = 10 \cdot \log_{10}\left(\frac{|S_{MPI}|}{|S_{MFH}|}\right) = 10 \cdot \log_{10}\left(\frac{|U_{MPI}|^2}{|U_{MFH}|^2}\frac{|Z_{MPI}|}{|Z_{MFH}|}\right)$$
$$= 20 \cdot \log_{10}\left(\frac{|U_{MPI}|}{|U_{MFH}|}\right) + 10 \cdot \log_{10}\left(\frac{|Z_{MPI}|}{|Z_{MFH}|}\right).$$

For the Impedances of the systems, the following relation must hold:

$$|Z_{MPI}| \geq |Z_{MFH}| \Rightarrow \frac{|Z_{MPI}|}{|Z_{MFH}|} \geq 1 \Rightarrow 10 \cdot \log_{10}\left(\frac{|Z_{MPI}|}{|Z_{MFH}|}\right) > 1.$$

It follows that

$$L_P = 20 \cdot \log_{10}\left(\frac{|U_{MPI}|}{|U_{MFH}|}\right) + 10 \cdot \log_{10}\left(\frac{|Z_{MPI}|}{|Z_{MFH}|}\right) \geq 20 \cdot \log_{10}\left(\frac{|U_{MPI}|}{|U_{MFH}|}\right) = L_U.$$

S3 – Supplementary Figures

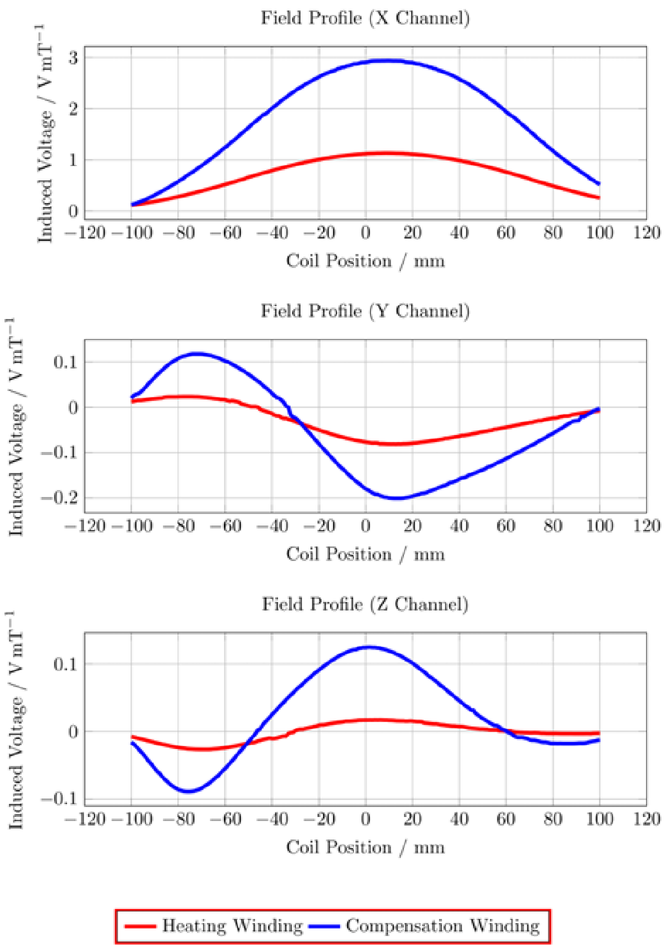

Figure S1: This figure shows the ratio of the induced voltage to the magnetic flux density for a single compensation turn (blue) and a single heating turn (red) translated along the bore, while the MPI system creates a magnetic field along a certain direction (x, y or z), using the winding diameters of single-coil MFH systems.

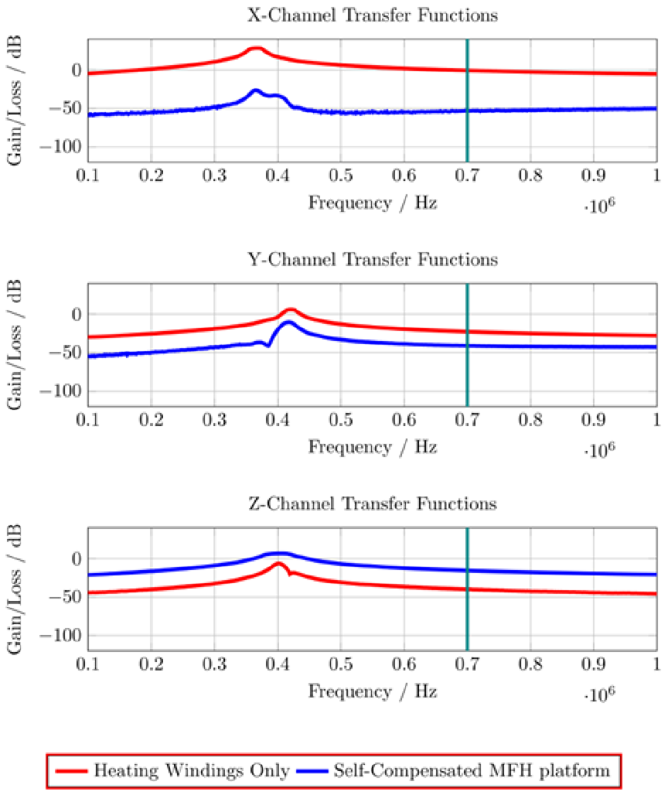

Figure S2: The transfer-functions of the heating winding and the self-compensated single-coil MFH system. The results show a decrease of overall transferred power. Even though z-channel a slight increase in transferred power, the power levels are low and especially for the frequency chosen for MFH marked in green the transferred power levels are on a sufficiently low level, since the gain/loss level is below the target of –16.75 dB for all channels.

S4 – Supplementary Tables

Table. S1: Electrical characteristics of the final MFH systems.

MFH system „Split-Coil “		MFH system „Single-Coil “	
Series Resistance	Inductance	Series Resistance	Inductance
165.05 mΩ	7.708 µH	44 mΩ	2.9 µH

Table. S2: Capacitor values for the impedance matching of the MFH systems.

MFH system „Split-Coil “		MFH system „Single-Coil “	
series capacitor	parallel capacitor	series capacitor	parallel capacitor
7.5 nF	79.26 nF	21.18 nF	153.2 nF

Table S3: Temperature data for the evaluation of the cooling capability

	MFH system „HIFU“			MFH system „BBB“		
power	temperature	duration	condition	temperature	duration	condition
10	21	600	stable	16	600	stable
20	21	600	stable	16	600	stable
30	21	600	stable	16	600	stable
40	26	600	stable	16	600	stable
50	29	600	stable	17	600	stable
75	36	600	stable	20	600	stable
100	43	600	stable	24	600	stable
125	48	600	stable	28	600	stable
150	54	600	stable	31	600	stable
175	60	420	unstable	34	600	stable
200	60	210	unstable	38	600	stable
225	60	180	unstable	37	300	stable
250	60	150	unstable	40	300	stable
275	60	120	unstable	42	300	stable
300	60	120	unstable	44	300	stable
325	60	120	unstable	47	300	stable
350	60	120	unstable	40	180	stable
375	-	-	-	42	180	stable
400	-	-	-	43	180	stable
425	-	-	-	44	180	stable
450	-	-	-	47	180	stable
475	-	-	-	50	180	stable
500	-	-	-	50	160	unstable
525	-	-	-	50	155	unstable
550	-	-	-	50	150	unstable
575	-	-	-	50	135	unstable
600	-	-	-	50	130	unstable
